# Hyperbaric oxygen therapy ameliorates intestinal and systematic inflammation by modulating dysbiosis of the gut microbiota in Crohn’s disease

**DOI:** 10.1186/s12967-024-05317-1

**Published:** 2024-05-30

**Authors:** Yong Li, Ruizheng Sun, Chen Lai, Kezhen Liu, Huixiang Yang, Ziheng Peng, Duo Xu, Fangling Huang, Keke Tang, Yu Peng, Xiaowei Liu

**Affiliations:** 1 Department of Gastroenterology, Xiangya Hospital, Central South University, Changsha, Hunan 410008 China; 2 Hunan International Scientific and Technological Cooperation Base of Artificial Intelligence Computer Aided Diagnosis and Treatment for Digestive Disease, Xiangya Hospital, Changsha, Hunan 410008 China; 3 Department of General Surgery, Xiangya Hospital, Central South University, Changsha, Hunan 410008 China; 4 Department of Microbiology and Molecular Genetics, Michigan State University, East Lansing, USA; 5 Department of Hyperbaric oxygen, Xiangya Hospital, Central South University, Changsha, Hunan 410008 China; 6 Research Center for Geriatric Disorder, Xiangya Hospital, Central South University, Changsha, Hunan 410008 China

**Keywords:** Hyperbaric oxygen therapy, Crohn’s disease, Gut microbiota, Inflammation, Fecal microbiota transplantation

## Abstract

**Background:**

Dysbiosis of the gut microbiota is pivotal in Crohn’s disease (CD) and modulated by host physiological conditions. Hyperbaric oxygen therapy (HBOT) is a promising treatment for CD that can regulate gut microbiota. The relationship between HBOT and the gut microbiota in CD remains unknown.

**Methods:**

CD patients were divided into an HBOT group (*n* = 10) and a control group (*n* = 10) in this open-label prospective interventional study. The fecal samples before and after HBOT were used for 16 S rRNA gene sequencing and fecal microbiota transplantation (FMT). A colitis mouse model was constructed using dextran sulfate sodium, and intestinal and systematic inflammation was evaluated. The safety and long-term effect of HBOT were observed.

**Results:**

HBOT significantly reduced the level of C-reactive protein (CRP) (80.79 ± 42.05 mg/L vs. 33.32 ± 18.31 mg/L, *P* = 0.004) and the Crohn’s Disease Activity Index (CDAI) (274.87 ± 65.54 vs. 221.54 ± 41.89, *P* = 0.044). HBOT elevated the declined microbial diversity and ameliorated the altered composition of gut microbiota in patients with CD. The relative abundance of *Escherichia* decreased, and that of *Bifidobacterium* and *Clostridium XIVa* increased after HBOT. Mice receiving FMT from donors after HBOT had significantly less intestinal inflammation and serum CRP than the group before HBOT. HBOT was safe and well-tolerated by patients with CD. Combined with ustekinumab, more patients treated with HBOT achieved clinical response (30%vs.70%, *P* = 0.089) and remission (20%vs.50%, *P* = 0.160) at week 4.

**Conclusions:**

HBOT modulates the dysbiosis of gut microbiota in CD and ameliorates intestinal and systematic inflammation. HBOT is a safe option for CD and exhibits a promising auxiliary effect to ustekinumab.

**Trial registration:**

Chinese Clinical Trial Registry, ChiCTR2200061193. Registered 15 June 2022, https://www.chictr.org.cn/showproj.html?proj=171605.

**Supplementary Information:**

The online version contains supplementary material available at 10.1186/s12967-024-05317-1.

## Introduction

Crohn’s disease (CD) is a chronic and relapsing inflammatory bowel disease (IBD) characterized by an impaired immune response, primarily affecting the gastrointestinal tract and exhibiting a progressive and destructive course [1]. CD is challenging to cure, 30% of patients requiring surgery within 5 years of diagnosis [2]. Therefore, new treatment methods to alleviate the burden of this disease are needed.

The gut microbiota, interacting with both the internal and external environments of the human body, plays a crucial role in the development of CD [3]. Numerous studies have extensively analyzed the characteristics and functional changes of the gut microbiota during CD onset, revealing dysregulation in its composition and functions under CD [4, 5]. The positive effects of fecal microbiota transplantation (FMT) in treating CD further underscore the importance of the gut microbiota [6, 7]. Collectively, the evidence suggests that interventions or modulation targeting the gut microbiota represent a promising direction for future CD treatments.

The composition of the gut microbiota is modulated by the oxygen. Alteration of host oxygenation could change the oxygen content of the luminal environment, suggesting that modulating the oxygenation of the gut could become a new approach for regulating the gut microbiota [8]. Hyperbaric oxygen therapy (HBOT) is a noninvasive treatment used in combination therapies for various diseases and is considered safe, having shown minimal side effects [9]. During HBOT, patients breathe 100% oxygen in a pressurized environment typically at 2.0-2.5 atmosphere absolute [10]. HBOT can improve depressive behavior in mice by reshaping the intestinal microbiota [11]. HBOT can enhance healing in ischemic colonic anastomosis by improving the local anaerobic environment [12]. Similarly, a few case reports have indicated that HBOT can improve clinical manifestations of IBD, although the underlying mechanisms have not been explored [13, 14]. Based on these findings, we hypothesize that HBOT will benefit patients with CD by modulating the composition of the host gut microbiota, thereby influencing the efficacy of biologic agents.

Therefore, we performed an open-label exploratory study to investigate the efficacy and safety of HBOT in CD patients. Omics analysis was employed to comprehensively understand the changes in the gut microbiota from before to after HBOT, followed by validation in an animal model to gain causal insights. Finally, a short-term follow-up was conducted to observe the impact of this therapy on the efficacy of subsequent biologic agents.

## Methods

### Patients and data collection

This trial was an open-label prospective interventional study and was conducted at the Xiangya Hospital Central South University, Changsha, Hunan Province, China. Patients were diagnosed by a multidisciplinary IBD team consisting of gastroenterologists, radiologists, and pathologists, following the guidelines of the Chinese Medical Association [15]. Patients aged 18 years or older with CD for at least 3 months were eligible for inclusion if they were in an active state with a baseline CD activity index (CDAI) score of 150–450. The treatment options for all patients were decided on after a comprehensive evaluation by the multidisciplinary team. All patients agreed to be treated with biologic agents. Patients with pregnancy, other concomitant diseases, or intolerance to ustekinumab were excluded from this trial. Patients were also not considered for this study if they had any disease contraindicating HBOT.

### Grouping and intervention

All patients enrolled in this trial met the indications for ustekinumab and were treated with it. At week 0, patients received a single intravenous infusion of ustekinumab, the dose of which depended on the weight of the patients [16]. The dose of 260 mg was given to patients weighing ≤ 55 kg, 390 mg was given to those weighing > 55 kg and ≤ 85 kg, and 520 mg was given to those weighing > 85 kg. At week 8, patients received maintenance therapy with 90 mg of ustekinumab subcutaneously at a frequency of every 12 weeks. Data were stored and collected in the electronic medical record system.

As this was an open-label exploratory study, all eligible patients were divided into an HBOT group and a control group based on their informed consent. Patients in the HBOT group were treated with 10 sessions of HBOT for 5 days, at two sessions a day, before starting ustekinumab treatment. HBOT was performed under 2.5 atm absolute (ATA) for 100 min (increased pressure for 20 min, ordinary pressure for 60 min and decreased pressure for 20 min), and the patients breathed 100% oxygen with four scheduled breaks to minimize the side effects. This treatment procedure was similar to that in a previous study and remained well within oxygen toxicity limits [17]. Equipment, such as hyperbaric oxygen chambers (YC2410-24, Hoto Oxygen Industrial, Shangdong, China) required for this treatment, was provided by Xiangya Hospital Central South University. Patients in the control group received the ustekinumab treatment routinely during hospitalization at week 0.

### Assessment of HBOT safety and short-term outcome

To evaluate the effectiveness of HBOT, the CDAI score, CRP level and fecal calprotectin level of patients before and after hyperbaric oxygen were compared. In terms of safety assessment, the supervising hyperbaric physician also evaluated the common side effects of HBOT, including complaints of trouble equalizing, visual changes and fatigue, in addition to assessing unsolicited adverse events after the treatment. The definition and quantification of side effects were based on Marvin’s review [18] to ensure accuracy and consistency.

### DNA extraction, PCR, and 16 S rRNA gene sequencing

Fecal DNA were extracted with the Thermo Scientific KingFisher Apex platform. The V3-V4 region of the bacterial 16 S rRNA gene was amplified by polymerase chain reaction (PCR) using the corresponding fusion primer reaction system (forward primer: 338 F ACTCCTACGGGAGGCAGCAG; reverse primer: 806R GGACTACHVGGGTWTCTAAT). Nucleic acid gel electrophoresis was conducted for quality inspection. Single-stranded cyclized products were produced through denaturation and circularization, while uncyclized linear DNA molecules were digested. Single-stranded circular DNA molecules were replicated via rolling cycle amplification, and a DNA nanoball (DNB) containing multiple copies of DNA was generated. Sufficient-quality DNBs were then loaded into patterned nanoarrays using a high-intensity DNA nanochip technique and sequenced through combinatorial probe-anchor synthesis (cPAS). Raw data were filtered to generate adequate quality clean reads by truncating reads with low-average-quality base pairs and removing reads with adapter contamination, ambiguous bases, or low-complexity. Operating taxonomic units (OTUs) were clustered with a 97% threshold by UPARSE using USEARCH (v7.0.1090) and chimeras were filtered by UCHIME (v4.2.40). Representative OUT sequences were aligned by the RDP classifier (v2.2) against the corresponding database for taxonomic annotations. Alpha diversity was measured by the Shannon index and Simpson index. Beta diversity analysis was conducted by software QIIME software (v1.80).

### Fecal microbiota transplantation

Before fecal microbiota transplantation (FMT), mice were fed with a cocktail of antibiotics (ampicillin 1 g/L, metronidazole 1 g/L, neomycin 1 g/L, and vancomycin 0.5 g/L) for 1 week to deplete microbiota, followed by wash-out period of 4 days to metabolize antibiotics before FMT, as reported in the previous literature [19]. Two patients’ (donors) feces before and after HBOT as donors were randomly selected for FMT. According to a previous study, 500 mg fresh stools from each donor before or after HBOT were weighed and homogenized by mixing with PBS buffer at a ratio of 100 mg/ml. After that, the mixture was centrifuged at 2500 × g for 5 min, and the supernatant was collected under anaerobic conditions. The prepared fecal samples were pooled and divided into the before HBOT (BH) group and the after HBOT (AH) group based on the different sources of stools. From 3 days before dextran sodium sulfate (DSS) intervention, the mice were colonized by oral gavage with 200 µl of BH or AH fecal slurry until the end of DSS intervention. The mice in each group were treated once daily.

### DSS-induced colitis

C57BL/6J male mice were induced with colitis by administering 2.5% DSS (36,000 to 50,000 molecular weight; MP Biomedicals) in their drinking water for 7 days. Each mouse received the same volume of liquid. The body weight and stool consistency were monitored daily [20]. The mice were euthanized on day 8. The tissue and feces were collected for subsequent experiments.

### Hematoxylin and eosin staining and histopathological analysis

The distal colon tissue was selected and fixed in 10% formalin solution, followed by embedding in paraffin. The tissue was then cut into 5-µm-thick sections and stained with hematoxylin and eosin (H&E) according to the standard protocols. Neutral balsam was used to cover the slides. According to published standards [20], histological blind scoring was assessed based on the degree of damage and inflammatory infiltration in the mucosal layer, submucosal layer, mucosal muscle layer, and serosal layer of the colon tissue. The total pathological score for each mouse was calculated.

### Real-time qPCR

According to the manufacturer’s instructions, RNA from mouse colon tissue was isolated with TRIzol reagent (TransGen), and reverse transcribed into cDNA with the PerfectStart Uni RT-qPCR Kit (TransGen). The DNA from mouse stool was extracted using the EasyPure Stool Genomic DNA Kit (TransGen). Real-time PCR (RT-PCR) was performed by using SYBR Green Master mix with ROX and an ABI QuantStudio 5 Flex instrument. The primers were listed in Table 1S. The gene expression was normalized to Gapdh. The specific genes were quantified to indicate the expression profile of the microbial community and normalized to total bacterial 16 S rRNA.

### Enzyme-linked immunosorbent assay

Serum samples were collected and centrifuged within one hour and stored at − 80 °C. The expression levels of COX2, PGE2 and CRP in serum were measured by enzyme-linked immunosorbent assay (ELISA) according to the manufacturer’s protocols (Cambridge, MA, U.S.A.).

### Observation of hysteresis effects of HBOT

To monitor the effects of HOBT on CD patients treated with ustekinumab, additional follow-up was conducted. As for safety assessment, in addition to the HBOT-related adverse effects mentioned earlier, any adverse reactions experienced by patients during ustekinumab administration following completion of HBOT should also be documented. The primary outcome was the clinical response at week 4, defined as a reduction of 70 points or more from the baseline in CDAI score [21]. The secondary outcomes included clinical response and endoscopic response at week 8, clinical remission and endoscopic remission at week 8, and the normalization of CRP and fecal calprotectin levels at weeks 4 and 8. Clinical remission was defined as a CDAI less than 150, and endoscopic remission was defined as an SES-CD score below 3. Endoscopic remission was considered when the SES-CD score fell by more than half from baseline [22]. The normalization of CRP was defined as less than 6 mg/L.

### Statistical analysis

Continuous variables, expressed as median and interquartile range, were analyzed using Student’s t test or nonparametric tests (Mean-Whitney test) for those not following a normal distribution. Categorical variables, such as clinical response and clinical remission, are expressed as n (%). Differences between categorical variables were analyzed using the chi-square test or Fisher’s exact probability test. All reported P values were two-tailed, and a value of *P* < 0.05 indicated statistical significance. The microbial variations and grouping significance of HBOT were tested using permutational MANOVA. The genera that differed in proportion before vs. after HBOT were identified with absolute fold change > 2 and adjusted *P* < 0.05 using the R package “DEseq2”. Correlations between the Shannon index, the level of CRP, and the relative abundance of significant genera were analyzed with the Spearman method. Linear regression models were constructed to evaluate the pairwise correlation. Estimates (Es) were extracted from the fitted linear regression models. The R package “mediation” was then used to perform casual mediation analysis. Average direct effect (ADE) was calculated to measure the direct effect of HBOT treatment on CRP level. Average causal mediated effect (ACME) was calculated to observe the mediated effect of Shannon index on CRP level. Analyses were performed with SPSS software (version 26.0) and R platform (4.2.1). For animal experiments, all analyses were performed with GraphPad Prism (version 9.0).

## Results

### Demographic characteristics of patients at baseline

**Fig. 1:**
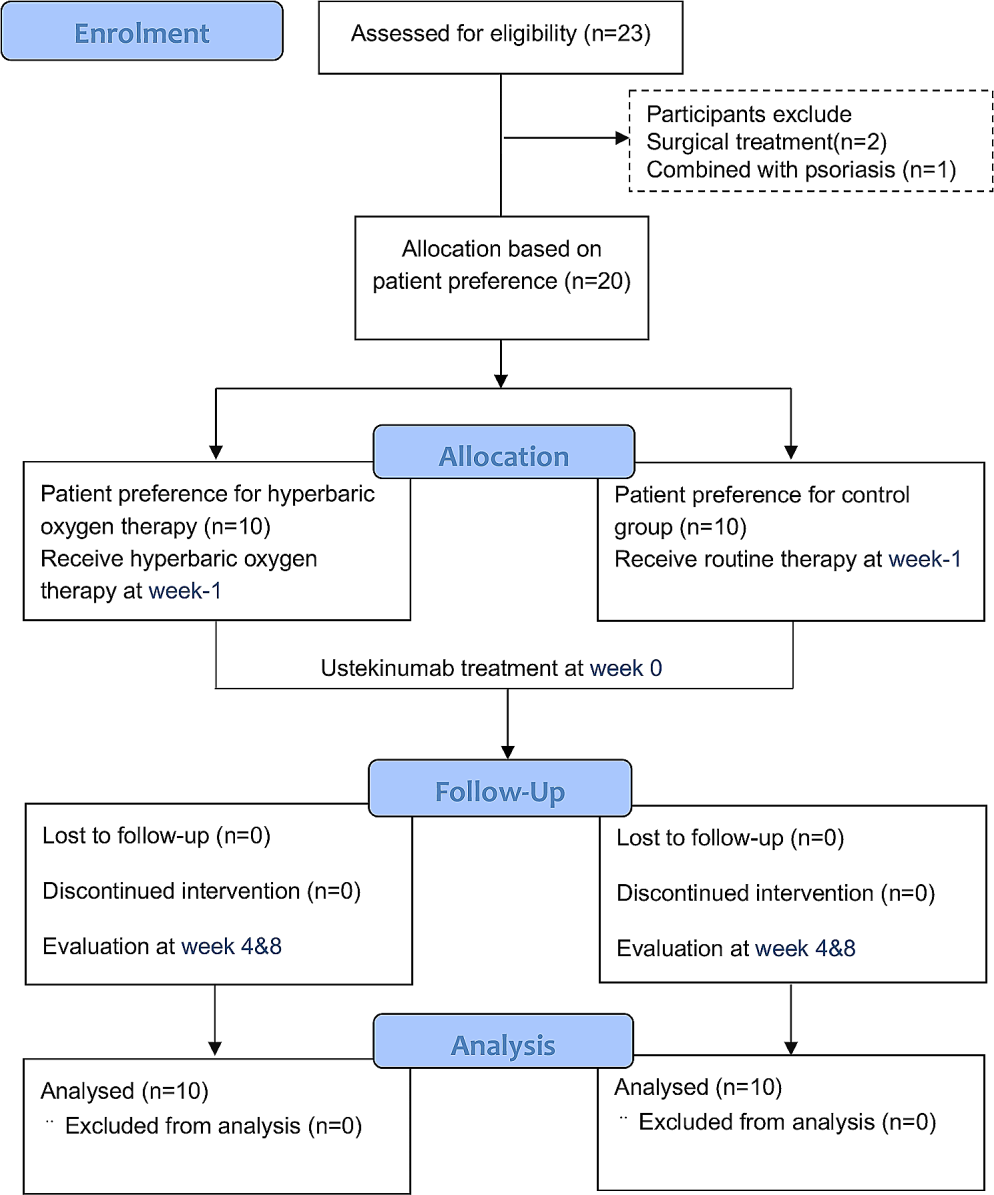
Flow of study recruitment, interventions and outcomes assessments

Twenty-three patients were recruited for the study, two of whom withdrew because they chose surgical treatment during the screening period, and one patient withdrew due to concomitant psoriasis. Enrolled patients in this study were divided into two groups according to whether they received HBOT, and a total of 20 patients were finally recruited (Fig. 1). The patients in the two groups were similar in demographic data (*P* > 0.05). In terms of laboratory tests, all enrolled patients had fecal calprotectin levels > 1800 µg/g. Patients in the HBOT group had higher levels of CRP than those in the control groups (80.79 ± 42.05 mg/L vs. 41.12 ± 40.61 mg/L, *P* = 0.046), but there were no significant differences in disease activity defined by the CDAI score (*P* = 0.140) or SES-CD score (*P* = 0.113). Table 1 displays the baseline characteristics of the study patients.

**Table 1:** Baseline characteristics of study patients

Variable	Control group(*n* = 10)	HBOT group(*n* = 10)	*P* value
Age, mean ± SD, years	31.92 ± 13.08	27.20 ± 7.26	0.332
BMI, mean ± SD, kg/m^2^	19.4 ± 2.2	18.6 ± 2.5	0.457
Male, n (%)	6(60%)	8(80%)	0.628
Location, n (%)			
L1	0(0%)	0(0%)	1.000
L2	4(40%)	2(20%)	0.314
L3	6(60%)	8(80%)	0.314
L4	0(0%)	0(0%)	1.000
Behavior, n (%)			
B1	3(30%)	3(30%)	1.000
B2	7(70%)	5(50%)	0.650
B3	0(0%)	2(20%)	0.474
Perianal fistulas, n (%)	6(60%)	8(80%)	0.628
Surgical history, n (%)	4(40%)	3(30%)	1.000
CDAI, mean ± SD	233.47 ± 53.84	274.87 ± 65.54	0.140
Serological examination			
WBC, mean ± SD, 10^9^/L	6.49 ± 1.91	7.38 ± 2.28	0.357
HGB, mean ± SD, g/L	120.42 ± 22.71	118.40 ± 15.78	0.819
PLT, mean ± SD, 10^9^/L	325.83 ± 116.34	437.00 ± 167.15	0.101
NLR, mean ± SD	4.42 ± 2.38	4.47 ± 1.77	0.958
PLR, mean ± SD	315.95 ± 193.91	291.96 ± 129.79	0.749
ALB, mean ± SD, g/L	31.6 ± 4.7	29.6 ± 4.3	0.334
D-D, mean ± SD, mg/L	0.17 ± 0.09	0.21 ± 0.10	0.359
ESR, mean ± SD, mm/h	61.75 ± 36.29	83.80 ± 19.30	0.107
CRP, mean ± SD, mg/L	41.12 ± 40.61	80.79 ± 42.05	0.046
FC, n (%)			1.000
>1800ug/g	10 (100%)	10 (100%)	
20ug/g- 1800ug/g	0 (0%)	0 (0%)	
≤20ug/g	0 (0%)	0 (0%)	
SES-CD, mean ± SD	10.8 ± 2.5	13.2 ± 3.8	0.113

*Abbreviation * BMI, body mass index; CDAI, Crohn’s disease activity index; WBC, white blood cell; HGB, hemoglobin; PLT, platelet; NLR, neutrophil-to-lymphocyte ratio; PLR, platelet-to-lymphocyte ratio; ESR, erythrocyte sedimentation rate; CRP, C-reactive protein; FC, fecal calprotectin; SES-CD, simple endoscopic score for Crohn’s disease; SD, standard deviation; HBOT, hyperbaric oxygen therapy

### The levels of CRP and CDAI score decreased after HBOT

After 10 sessions of HBOT, the levels of CRP (80.79 ± 42.05 mg/L vs. 33.32 ± 18.31 mg/L, *P* = 0.004) and the CDAI (274.87 ± 65.54 vs. 221.54 ± 41.89, *P* = 0.044) decreased significantly from baseline among the patients in the HBOT group, but the erythrocyte sedimentation rate (ESR) did not decrease (*P* = 0.251). A decrease in fecal calprotectin levels was observed in three patients after hyperbaric oxygen, in one of whom it returned to the normal level. There was also a mild decrease in the serum levels of hemoglobin, although this decrease was not statistically significant (*P* > 0.05), as shown in Table 2.

**Table 2:** Comparison of disease status before and after 10 sessions of HBOT

Variable	Before HBOT(*n* = 10)	After HBOT(*n* = 10)	*P* value
CDAI, mean ± SD	274.87 ± 65.54	221.54 ± 41.89	0.044
Serological examination			
WBC, mean ± SD, 10^9^/L	7.38 ± 2.28	7.47 ± 2.30	0.930
HGB, mean ± SD, g/L	118.40 ± 15.78	108.80 ± 11.71	0.139
PLT, mean ± SD, 10^9^/L	437.00 ± 167.15	416.80 ± 149.16	0.779
NLR, mean ± SD	4.47 ± 1.77	3.69 ± 1.10	0.252
PLR, mean ± SD	291.96 ± 129.79	294.43 ± 80.74	0.959
ALB, mean ± SD, g/L	29.6 ± 4.3	31.8 ± 5.3	0.322
D-D, mean ± SD, mg/L	0.21 ± 0.10	0.16 ± 0.07	0.212
ESR, mean ± SD, mm/h	83.80 ± 19.30	72.40 ± 23.50	0.251
CRP, mean ± SD, mg/L	80.79 ± 42.05	33.32 ± 18.31	0.004
FC, n (%)			0.171
>1800ug/g	10 (100%)	7 (70%)	
200ug/g- 1800ug/g	0 (0%)	2 (20%)	
≤200ug/g	0 (0%)	1 (10%)	

*Abbreviation *CDAI, Crohn’s disease activity index; WBC, white blood cell; HGB, hemoglobin; PLT, platelet; NLR, neutrophil-to-lymphocyte ratio; PLR, platelet-to-lymphocyte ratio; ESR, erythrocyte sedimentation rate; CRP, C-reactive protein; FC, fecal calprotectin; SES-CD, simple endoscopic score for Crohn’s disease; SD, standard deviation; HBOT, hyperbaric oxygen therapy

Considering the safety of HBOT, the side effects of HBOT include complaints of trouble equalizing, visual changes and fatigue. No patients experienced hyperbaric oxygen-related adverse events, and no patients were unable to tolerate the 10 sessions.

### The diversity and composition of the gut microbiota changed after HBOT

**Fig. 2:**
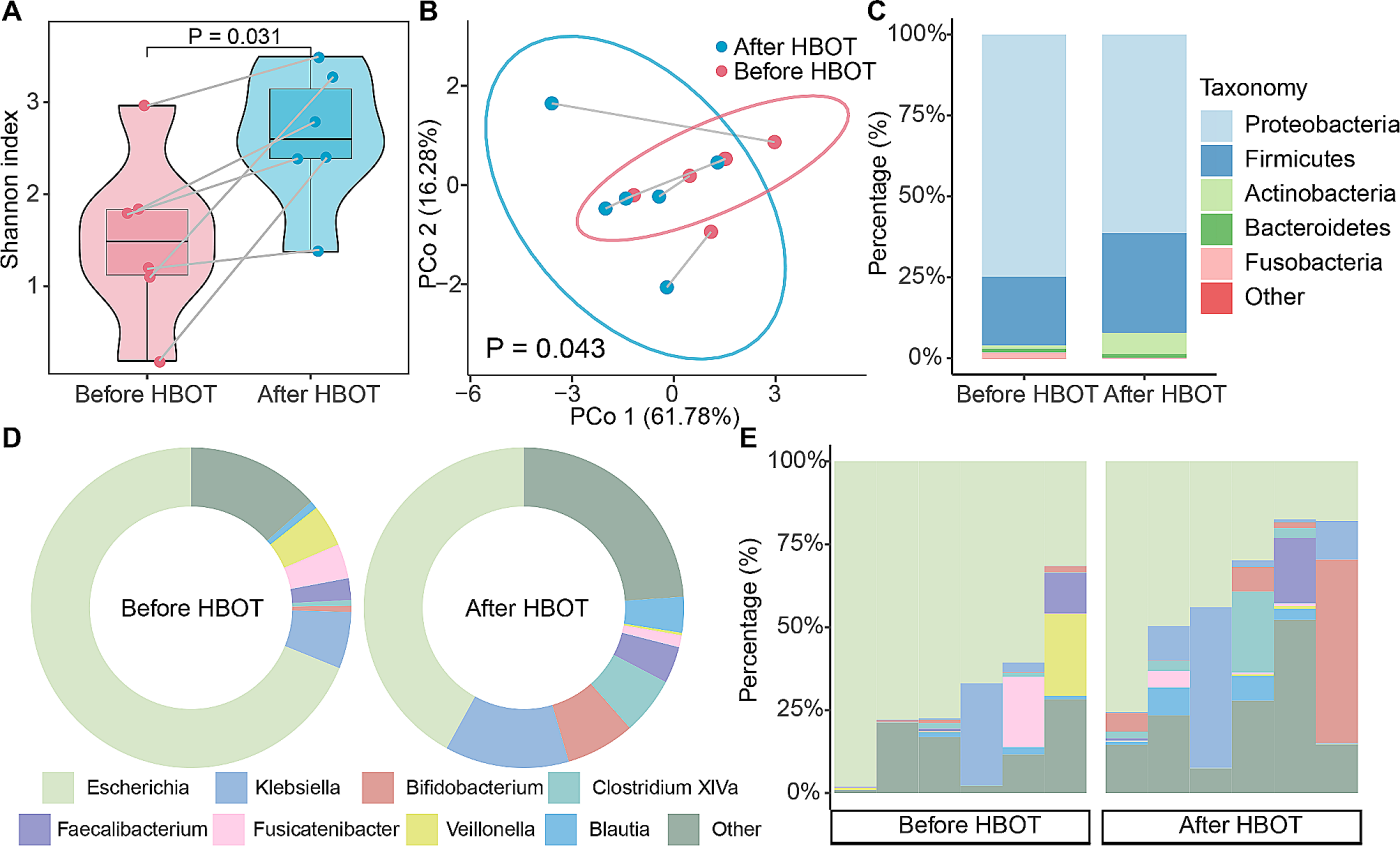
Diversity and composition of gut microbiota in patient with CD before and after HBOT. **(A)** The boxplot showed the corresponding change of Shannon index in patients with CD before and after HBOT. **(B)** Permutational multivariate analysis of variance (Adonis) showed that the composition of the gut microbiota was significantly altered among patients after HBOT (*P* = 0.043). **(C)** The stack plot of average relative abundance in the group of before HBOT and after HBOT at phylum level. **(D)** The circle plot exhibited the compositional differences of genera before and after HBOT. **(E)** The stack plot of each sample showed the taxonomic composition in individuals with CD. CD, Crohn’s disease; HBOT, hyperbaric oxygen therapy

We then explored the alterations in the gut microbiota in patients from before to after HBOT. After quality testing, the fecal samples from six pairs of patients met the requirements. The Shannon index of the gut microbiota in patients after HBOT was significantly higher than that in the same patients before HBOT (*P* = 0.031, Fig. 2A). Similar notable changes in alpha diversity were also observed with Simpson’s index (Figure S1). The composition of the gut microbiota was significantly altered among patients after HBOT (*P* = 0.043), and the variations in matched samples before and after HBOT were distinguished by the first principle coordinate axis (Fig. 2B).

The averages relative abundance of Proteobacteria decreased from 74.8 to 61.3% after HBOT treatment. The relative abundance of Firmicute and Actinobacteria increased by 9.5% and 5.5%, respectively. Fusobacteria dropped from 1.8% before HBOT to 0.1% after HBOT (Fig. 2C). At the genus level, the average relative abundance of *Escherichia* decreased from 67.8 to 34.9% after HBOT. The relative abundance of *Bifidobacterium*, *Klebsiella*, and *Clostridium XIVa* rose by 5.2%, 4.8%, and 4.2%, respectively (Fig. 2D). The dominant Escherichia, with over 50% relative abundance in five out of six patients with CD, dropped its abundance to less than 50% in four out of the six after HBOT. (Fig. 2E).

### Significant microbial diversity mediated the causal relationship between HBOT and CRP

**Fig. 3:**
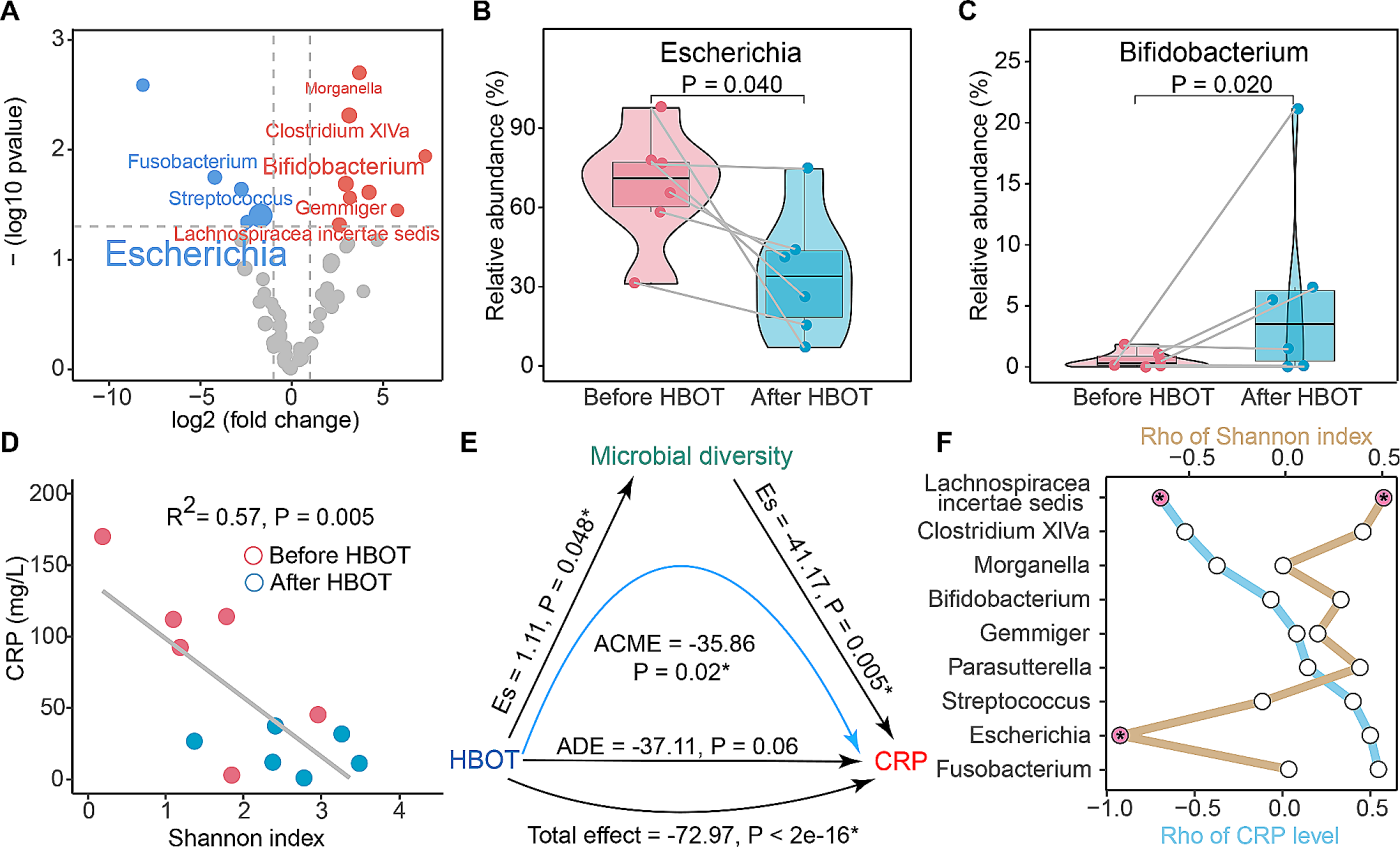
Significant genera and the relationship between HBOT, gut microbiota, and CRP. (**A**) The volcano plot revealed the significantly differential genera after HBOT. The size of genus name represented the average relative abundance in CD. Genera in blue meant depletion and genera in red meant enrichment after HBOT. The boxplot showed the change of *Escherichia* (**B**) and *Bifidobacterium* (**C**) in corresponding samples before and after HBOT. (**D**) The scatter plot and linear regression exhibited a significant negative correlation between Shannon index and the level CRP. (**E**) The plot demonstrated the correlation and casual relationships among HBOT, microbial diversity, and the level of CRP. (**F**) The plot reveals the coefficients between the relative abundance of significant genera and Shannon index (in brown) / the level of CRP (in blue). Plot in red with a “*” means P value < 0.05 in Spearman test. CRP, C-reactive protein; HBOT, hyperbaric oxygen therapy

A total of 18 significantly differential genera were identified, with 11 enriched and seven depleted after HBOT. Among genera with relative abundance > 0.5%, *Escherichia*, *Fusobacterium*, and *Streptococcus* markedly reduced after HBOT, and five genera increased after HBOT, including *Lachnospiracea incertae sedis*, *Gemmiger*, *Bifidobacterium*, *Clostridium XIVa*, and *Morganella* (Fig. 3A). *Escherichia* was the leading genus in patients with CD, with a maximum decrease of up to 90.8% after HBOT (Fig. 3B). *Bifidobacterium*, with the highest relative abundance enriched in after HBOT, notably increased by 21.0% at most (Fig. 3C).

We then explored the relationship between gut microbiota and the level of CRP. With the decrease in CRP after HBOT, the Shannon index of the gut microbiota in patients with CD increased. There was a significant negative linear correlation between the CRP level and Shannon index (R^2^ = 0.57, *P* = 0.005, Fig. 3D). There were also notable relationships between HBOT, the Shannon index of gut microbiota, and the level of CRP. Through causal mediation analysis, we found that HBOT had a strong total effect on down-regulating the level of CRP. The average direct effect of HBOT on CRP might not be of important (*P* = 0.06), and the average causal mediation effects of HBOT on CRP mediating the Shannon index were marked (*P* = 0.02, Fig. 3E). This indicates that ten sessions of HBOT significantly reduce the level of CRP in patients with CD, and gut microbial diversity plays an essential role in mediating this effect.

With an increase in the correlation coefficient between significant differential genera after HBOT and the level of CRP, the rho coefficient between these genera and the Shannon index exhibited a declining trend. *Lachnospiracea incertae sedis* had a notably positive correlation with the Shannon index and a negative correlation with the level of CRP. The rho between the *Escherichia* and Shannon index remarkably reached − 0.92, suggesting that the relative abundance of the *Escherichia* served as a negative indicator for the Shannon index (Fig. 3F).

### Fecal microbiota transplantation from gut microbiota after HBOT alleviates colitis in mice

**Fig. 4:**
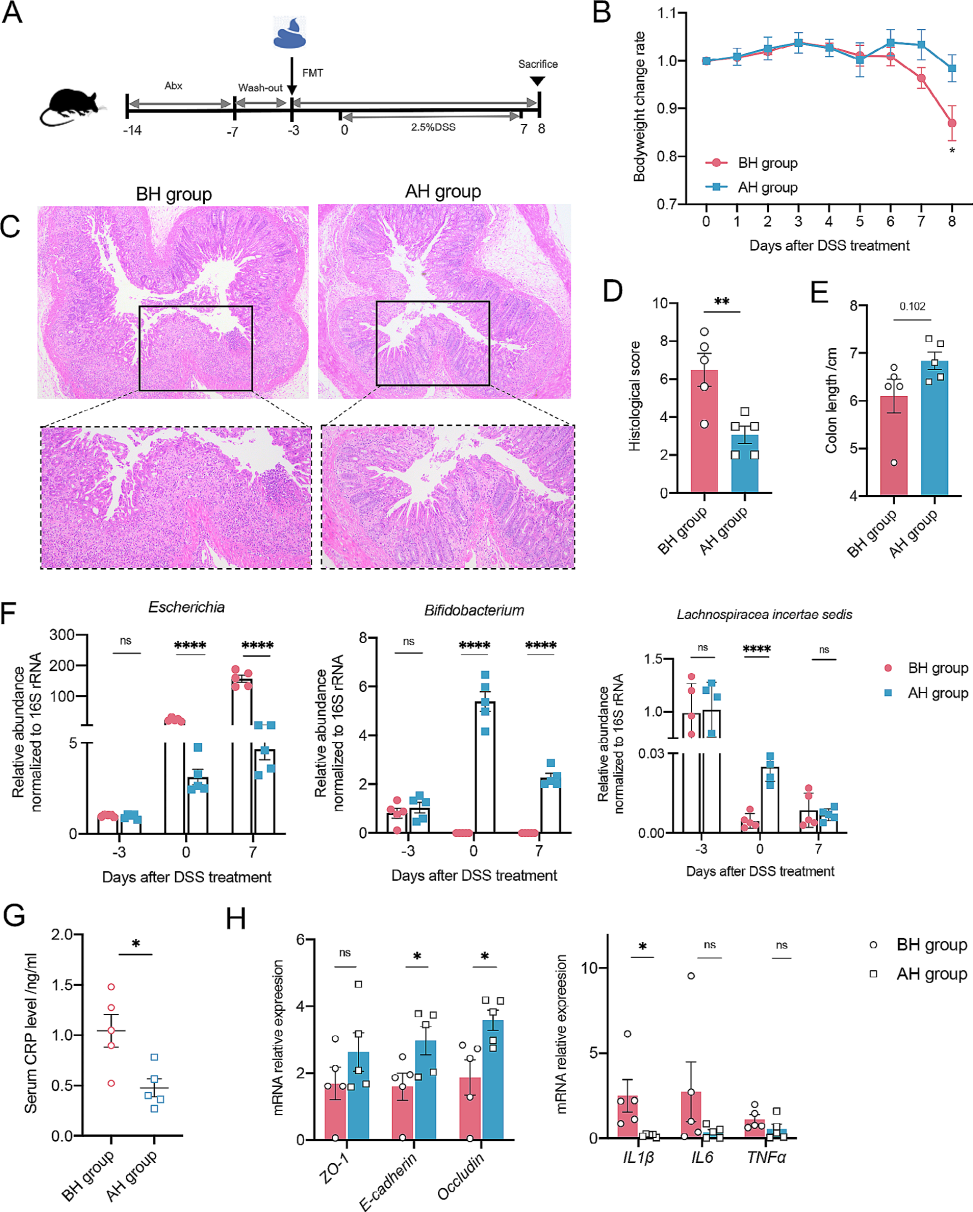
Fecal microbiota transplantation from gut microbiota after HBOT alleviates colitis in mice. (**A**) The experimental design showing an FMT from human donors to mice. (**B**) Body weights shown as percentage of starting weight (*n* = 5 per group) and colon length (**E**). (**C**) Representative images of distal colon stained with H&E and histological score was calculated (**D**). (**F**) Time-course changes in the relative abundance of *Escherichia, Bifidobacterium*, and *Lachnospiracea incertae sedis*. (**G**) Quantification of serum levels of CRP. (**H**) Expression levels of genes involved in tight junction protein and inflammation. Data points represent individual mice. All data are represented as means ± SEM. p values were calculated by Student’s t test. For (**B**), (**F**) Student’s t test was performed independently at each time point; ∗*p* < 0.05, ∗∗*p* < 0.01, and∗∗∗*p* < 0.001. FMT, Fecal microbiota transplantation; H&E, Hematoxylin and eosin staining; CRP, C-reactive protein

To investigate the potential causal relationship between the widespread gut microbiota alteration induced by HBOT and the reduction of inflammation in patients with CD, a FMT was performed into conventional antibiotic-treated mice fed with DSS (Fig. 4A). The fecal samples used in FMT were from enrolled patients before or after HBOT. Compared with the BH group, FMT from the AH group resulted in significantly attenuated weight loss and increased colon length (Fig. 4B and E). Histological assessments showed much lower inflammatory cell infiltration, mucosal damage, and overall histology score in the colon after FMT form the AH group (Fig. 4C and D). Additionally, the AH group had a lower level of CRP in the serum (Fig. 4G). Further analysis revealed that the fecal samples from mice receiving FMT form AH fecal samples had a lower relative abundance of *Escherichia* and a higher relative abundance of *Bifidobacterium* on day 7 of DSS intervention than the BH group, but there were no significant differences in *Lachnospiracea incertae sedis* (Fig. 4F). At the molecular level, the expression of genes encoding inflammatory proteins was reduced after receiving the FMT from AH samples (Fig. 4H). There were increased expression of genes related to tight junctions in the AH groups, suggesting that the destruction of gut barrier integrity was relatively light (Fig. 4H).

### HBOT shows a trend toward improving clinical outcomes in patients treated with ustekinumab at week 4

After treatment with ustekinumab at week 4, a higher proportion of patients treated with HBOT achieved clinical response (30% vs. 70%, *P* = 0.089) and remission (20% vs. 50%, *P* = 0.160) than patients in the control groups. However, both groups showed similar proportions of clinical response (60% vs. 80%, *P* = 0.628) and remission (50% vs. 70%, *P* = 0.325) at week 8. When considering endoscopic remission and endoscopic response, both groups showed similar patient distributions at 8 weeks (*P* > 0.05).

**Fig. 5:**
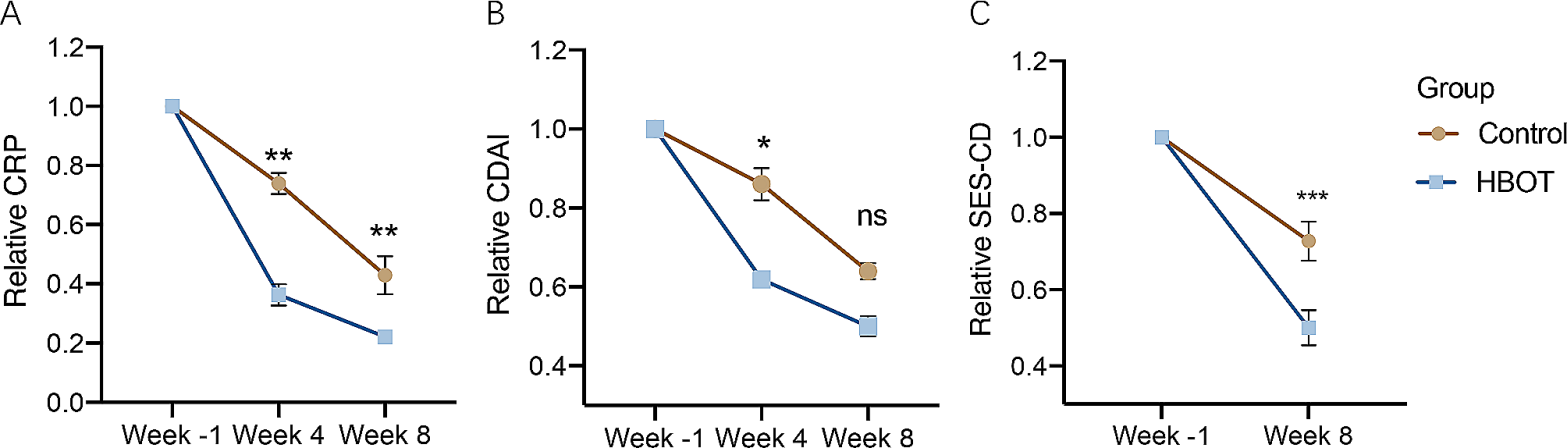
Time-course changes in the variables assessed for endpoints between control group and HBOT group. Graphs show the relative changes of CRP level (**A**), CDAI (**B**) and SES-CD (**C**) at different time points (*n* = 10 per group). The relative decline rates at week 4 and/or week 8 compared to baseline were tested by Student’s t test (*n* = 10 per group), ∗*p* < 0.05, ∗∗*p* < 0.01, and ∗∗∗*p* < 0.001. CDAI, Crohn’s disease activity index; CRP, C-reactive protein; SES-CD, simple endoscopic score for Crohn’s disease; HBOT, hyperbaric oxygen therapy

There were no significant differences in the CDAI score or CRP levels between the two groups, either at week 4 or at week 8 (Figure S2). However, when considering the relative change rate of the above parameters, there were greater differences in these two groups (Fig. 5), ignoring the situation where the control group did not receive sham hyperbaric air. At week 4, patients exposed to hyperbaric oxygen exhibited a trend toward higher rates of normalization in CRP (20% vs. 50%, *P* = 0.160) as well as fecal calprotectin (20% vs. 50%, *P* = 0.126), but at week 8, the rates in both groups remained similar (Table 3).

**Table 3:** Comparison of clinical outcomes with different treatments

Variable	Control group(*n* = 10)	HBOT group(*n* = 10)	*P* value
Week 4			
CDAI, mean ± SD	197.67 ± 63.71	188.87 ± 51.21	0.738
CRP, mean ± SD, mg/L	30.19 ± 16.00	25.86 ± 22.40	0.875
CRP normalization, n (%)	2 (20%)	5 (50%)	0.160
FC, n (%)			0.129
>1800ug/g	5 (40%)	1 (10%)	
200ug/g- 1800ug/g	3 (40%)	4 (40%)	
≤200ug/g	2 (20%)	5 (50%)	
Clinical response, n (%)	3 (30%)	7(70%)	0.089
Clinical remission, n (%)	2(20%)	5(50%)	0.160
Week8			
CDAI, mean ± SD	160.52 ± 75.99	146.05 ± 64.34	0.651
CRP, mean ± SD, mg/L	16.08 ± 17.91	15.9 ± 17.52	0.988
CRP normalization, n (%)	7(70%)	7(70%)	1.000
FC, n (%)			0.476
>1800ug/g	1 (10%)	0 (0%)	
200ug/g- 1800ug/g	3(30%)	2(20%)	
≤200ug/g	6(60%)	8 (80%)	
SES-CD, mean ± SD	8.6 ± 5.9	7.2 ± 4.8	0.568
Clinical response, n (%)	6(60%)	8(80%)	0.628
Clinical remission, n (%)	5 (50%)	7 (70%)	0.325
Endoscopic response, n (%)	3 (30%)	3 (30%)	1.000
Endoscopic remission, n (%)	1 (10%)	2(20%)	0.500

*Abbreviation *CDAI, Crohn’s disease activity index; CRP, C-reactive protein; FC, fecal calprotectin; SES-CD, simple endoscopic score for Crohn’s disease; SD, standard deviation; HBOT, hyperbaric oxygen therapy

Nor did any patient experience treatment-related discomfort during the 8-week follow-up period after taking ustekinumab.

### Hyperbaric oxygen therapy ameliorates the inflammatory cascade by regulating COX2/PGE2

Furthermore, we conducted a preliminary investigation of the molecular mechanisms associated with the effects of HBOT in patients with CD. We found that the protein expression of COX2 was significantly decreased by hyperbaric oxygen exposure (0.080 ± 0.003 ng/ml vs. 0.076 ± 0.002 ng/ml, *P* = 0.014, Figure S3A). Similarly, PGE2, a key inflammatory mediator generated catalytically by COX2, showed the same trend after hyperbaric oxygen treatment (3185.2 ± 389.8 pg/ml vs. 2778.6 ± 364.0 pg/ml *P* = 0.029, Figure S3B). This result suggested that hyperbaric oxygen may inhibit the occurrence of the inflammatory cascade by regulating the expression of COX2/PGE2.

## Discussion

The available therapies for patients with CD are complex due to the various disease characteristics. The reduction in diversity and the altered gut microbial composition plays a pivotal role in the dynamics of CD and emerge as an outstanding potential target for CD treatment [23, 24]. We found that the clinical application of HBOT significantly downregulated CRP and lowered the CDAI in patients with CD. HBOT also mitigated the altered composition of gut microbiota and improved the reduced microbial diversity found in CD. By modulating the gut microbiota, HBOT reduced the serum level of CRP and intestinal inflammation. Prospectively, HBOT combined with ustekinumab improved the efficiency of agents without increasing adverse reactions. An initial exploration of the potential mechanism indicated that HBOT may inhibit the COX2/PGE2 to ameliorate the inflammation, thereby exerting a rapid effect on patients with CD.

Although the pathogenesis of CD remains unclear, many studies have demonstrated that the inflammatory response is involved in the development and progression of the disease [25]. Recent studies have emphasized that early control of inflammation enhances the response to agents and leads to a reduction in complications [26]. Interestingly, our study found that the decrease in CRP was statistically significant after HBOT. This result suggested that HBOT might play a role in reducing the inflammatory load, which has been reported in other studies. However, the ESR, another indicator of reactive inflammation levels, was not significantly decreased. This may be because the ESR was slow to react to positive acute-phase reactants, resulting in slow recognition of the inflammatory response, let alone minor inflammation [27]. Conversely, CRP, with its faster kinetics and a shorter half-life, can decrease rapidly upon inflammation resolution, allowing for monitoring of the treatment response [28]. In addition, CRP has been increasingly used as a secondary endpoint in CD clinical trials, as an increasing number of studies have found that combining biomarker and CDAI-defined endpoints may improve the trial efficiency by increasing the separation of response rates in different intervention groups [29]. Therefore, CRP has been used in over 50 clinical trials as a biomarker of activity in CD [30]. The above results demonstrated that HBOT has good anti-inflammatory effects.

HBOT significantly ameliorated the dysbiosis of the gut microbiota in CD patients. The gut microbiota, as a complex and intensively populated community, is located in an environment determined by host physiological status [8]. HBOT to the host increases oxygenation from intestinal tissue to the lumen, and the composition of the gut microbiota can be altered by directly affecting microbes and by regulating the host immune system [31, 32]. In our study, the relative abundance of predominant *Escherichia* in patients with CD markably declined after HBOT. As a facultative anaerobic genus, the growth of *Escherichia* was inhibited under hyperbaric oxygen, according to ex vivo experiments [33]. The relative abundance of Enterobacteriaceae in HBOT treated mice was significantly lower than that in controls [8]. With the decline in *Escherichia*, more ecological niches became available and the microbial diversity significantly rose. The relative abundance of other genera increased, mainly including *Bifidobacterium*, *Klebsiella*, and *Clostridium XIVa*. However, the anaerobic or aerobic nature of microbes does not represent the trend of the population in their host after HBOT. More attention should be paid to the dynamic pattern of gut microbiota after HBOT in health and diseases.

As one of the leading hallmarks in CD, microbial dysbiosis is characterized by lower reduction and altered composition [34]. Different microbial signatures have been established in various observational studies, and *Escherichia* is one of the most pivotal genera enriched in CD [35–37]. In our CD cohort, *Escherichia* occupied over 50% of the relative abundance in five of the six CD patients before HBOT and had a strong linear correlation with microbial diversity. The onset and relapse of CD was suggested to correlate with the systemic immune response to different bacterial events and the level of anti-*E. coli* was positively correlated with serum inflammatory cytokines including IL6 and IFNα [38]. Mesenteric fat plays a significant role in connecting *E. coli* with the immunological activity of patients with CD. A high abundance of *Escherichia* might cause bacterial translocation to mesenteric fat and trigger adipocytes to produce more CRP [39]. The decreases in *Bifidobacterium* and *Clostridium XIVa* are a part of the characteristics of dysbiosis in CD as well [34]. *Bifidobacterium longum* has been demonstrated to have the capacity to relieve colitis and the potential to be an auxiliary treatment [40]. *Clostridium XIVa* has been strongly correlated with the intestinal metabolism of deoxycholic acid, and the level of bile acid metabolism was also significantly reduced in patients with CD compared with healthy controls [41]. *Klebsiella* was found elevated after HBOT in our study, but the trend was not significant. The *Klebsiella* is a potential pathobiont in CD, and a clade of *Klebsiella pneumoniae* strains could enhance intestinal inflammation [42]. Dysbiosis even contributed to the pathogenesis and activity of CD, and the situation was improved after HBOT.

HBOT ameliorated intestinal inflammation by regulating the gut microbiota. In this study, we performed causal mediation analysis and FMT to identify the causal relationship between HBOT, gut microbiota and inflammation. Consistent with the traits of their human FMT donors, the mice receiving FMT from AH samples exhibited a higher relative abundance of *Bifidobacterium* and a lower proportion of *Escherichia* compared with the BH group. In the AH groups, the expression level of tight-junction genes was upregulated, and the gene expression of inflammation was downregulated. *Escherichia* has the potential to destroy gut barrier integrity, cause bacterial translocation, and induce systematic inflammation [39, 43]. By contrast, the *Bifidobacterium* produces short-chain fat acids, improves intestinal barrier functions, prevents bacterial translocation, and reduces inflammation [44, 45]. These bacteria play a pivotal role in the mediating the protective function of HBOT, which was verified by the fact that mice receiving FMT from the AH group had significantly lower serum CRP and histology scores in intestinal tissue.

In addition to gut microbiota mediation, there might be other mechanisms underlying the treatment effect of HBOT in CD. There has been little research on the mechanism by which hyperbaric oxygen improves diseases [46]. Based on the observations of our study in the clinical phenomenon, a preliminary exploration was performed. Through the detection of related proteins in the serum of patients before and after hyperbaric oxygen treatment, we found that hyperbaric oxygen could effectively reduce the expression of COX2, which was the key link causing the subsequent inflammatory response. COX2 is regarded as an inducible enzyme which catalyzes the generation of prostaglandins [47]. The expression of COX2 was low in normal tissue cells, but elevated 10 to 80 folds in inflammatory states, resulting in an increase in PGE2 [48]. PGE2, a proinflammatory mediator, is significantly increased during CD. PGE2 plays a positive auxiliary role in TNFα activation in macrophages by regulating intracellular cAMP levels [49]. Our results confirmed that HBOT reduced the expression of COX2 and PGE2, which may decrease the occurrence of the inflammatory cascade in vivo. Early resolution of inflammation may be beneficial to the efficacy of ustekinumab [25]. Therefore, we speculate that hyperbaric oxygen therapy may inhibit the expression of COX2/PGE2, thereby reducing the inflammatory load at the early stage of treatment and effectively promoting the onset of action in ustekinumab treatment.

Although ustekinumab has shown promising therapeutic effects in CD, the onset of action is modest [50]. We found that a higher proportion of patients achieved clinical response and remission at week 4 after hyperbaric oxygen exposure. This may be because hyperbaric oxygen therapy inhibits the continued production of inflammatory factors, allowing more ustekinumab to neutralize interleukins.However, whether the impact of HBOT will affect long-term clinical outcomes still requires time for continuous follow-up. Interestingly, our subsequent follow-up also demonstrated that additional HBOT does not increase the side effects of ustekinumab, but this requires a larger cohort to verify.

Currently, there are still many non-traditional therapies being discovered for patients with CD. Stem cell transplantation has been proven to be effective in treating CD, but it is difficult to operate and expensive [51, 52]. FMT is also an emerging treatment [53, 54], but it depends on stool banks and certain medical technologies [55]. In this study, HBOT was found to have a positive effect on the relief of CD. This therapy is easily accessible and operable, making it a viable option for hospitals with limited resources. While the efficacy of HBOT may be limited, its easy accessibility allows for wider promotion and application, ultimately benefiting more potential patients compared to certain non-traditional methods.

There were some limitations in the study. First, due to the groundbreaking exploratory nature of the research, the sample size was small. There may be some bias in inclusion. Patients with more severe diseases may be more inclined to choose HBOT treatment, which also leads to higher baseline CRP levels in the HBOT group. Therefore, the results should be regarded as evidence that a clinical benefit of HBOT may exist to guide further trial designs. Meanwhile, the patients in control group did not receive sham hyperbaric air and lacked detection of microbial composition. In future prospective studies, we will further balance the baseline characteristics of the two groups and address these shortcomings to further verify our results. Second, patients with CD in our study had a high relative abundance of *Escherichia* before HBOT and exhibited an increase in *Bifidobacterium* and *Clostridium XIVa* after HBOT. The specific functions of these genera should be clarified, and the interactions between the gut microbiota and host during the process of HBOT should be further explored. Finally, this study focused on the mediating effect of the gut microbiota in HBOT treatment. Other mechanisms of HBOT in patients with CD should be further explored, especially the cellular and molecular mechanisms in the host. More in-depth studies need to be performed at the animal and cellular levels.

## Conclusion

Hyperbaric oxygen therapy ameliorates intestinal and systematic inflammation by modulating dysbiosis of the gut microbiota in CD. Hyperbaric oxygen has potential benefits for patients with CD undergoing ustekinumab treatment. Nonetheless, high-quality phase 2 clinical studies and basic research are still needed for verification.

### Electronic supplementary material

Below is the link to the electronic supplementary material.

Supplementary Material 1

Supplementary Material 2

## Declarations

### Data sharing statement

All data included in this study are available upon request by contact with the corresponding author.

## Abbreviations

CD: Crohn’s disease
IBD: Inflammatory bowel disease
ATA: Atm absolute
PCR: Polymerase chain reaction
OTUs: Operating taxonomic units
FMT: Fecal microbiota transplantation
BMI: Body mass index
CDAI: Crohn’s disease activity index
WBC: White blood cell
HGB: Hemoglobin
PLT: Platelet
NLR: Neutrophil-to-lymphocyte ratio
PLR: Platelet-to-lymphocyte ratio
ESR: Erythrocyte sedimentation rate
CRP: C-reactive protein
FC: Fecal calprotectin
SES-CD: Simple endoscopic score for Crohn’s disease
SD: Standard deviation
HBOT: Hyperbaric oxygen therapy
