# Supplementary material for: Hyperbaric oxygen therapy ameliorates intestinal and systematic inflammation by modulating dysbiosis of the gut microbiota in Crohn’s disease
Source: J Transl Med. 2024 May 30;22:518. doi: 10.1186/s12967-024-05317-1 (PMC11137967; doi:10.1186/s12967-024-05317-1)
Supplement: Supplementary file 1 — Supplementary Material 1 [file 12967_2024_5317_MOESM1_ESM.docx]

**Table S1 Primer sequences of RT-PCR**

|  | Forward Sequence | Reverse Sequence |
| --- | --- | --- |
| 16S rRNA | ATTACCGCGGCTGCTGGC | ACTCCTACGGGAGGCAGCAGT |
| *Escherichia* | CAACGAACTGAACTGGCAGA | CATTACGCTGCGATGGAT |
| *Bifidobacterium* | CGGGTGAGTAATGCGTGACC | TGATAGGACGCGACCCCA |
| *Lachnospiracea incertae sedis* | CCTGACTAAGAAGCTCCGGC | CAAAAGCAGTTCCGGGGTTG |
| Zo1 | GCCGCTAAGAGCACAGCAA | GCCCTCCTTTTAACACATCAGA |
| E-cadherin | CAGGTCTCCTCATGGCTTTGC | CTTCCGAAAAGAAGGCTGTCC |
| Occludin | CCTCCAATGGCAAAGTGAAT | CTCCCCACCTGTCGTGTAGT |
| IL1β | GAGAGCCGGGTGACAGTATC | TGACAAACTTCTGCCTGACG |
| IL6 | AGTTGCCTTCTTGGGACTGA | CAGAATTGCCATTGCACAAC |
| TNFα | CGTCAGCCGATTTGCTATCT | CGGACTCCGCAAAGTCTAAG |

**
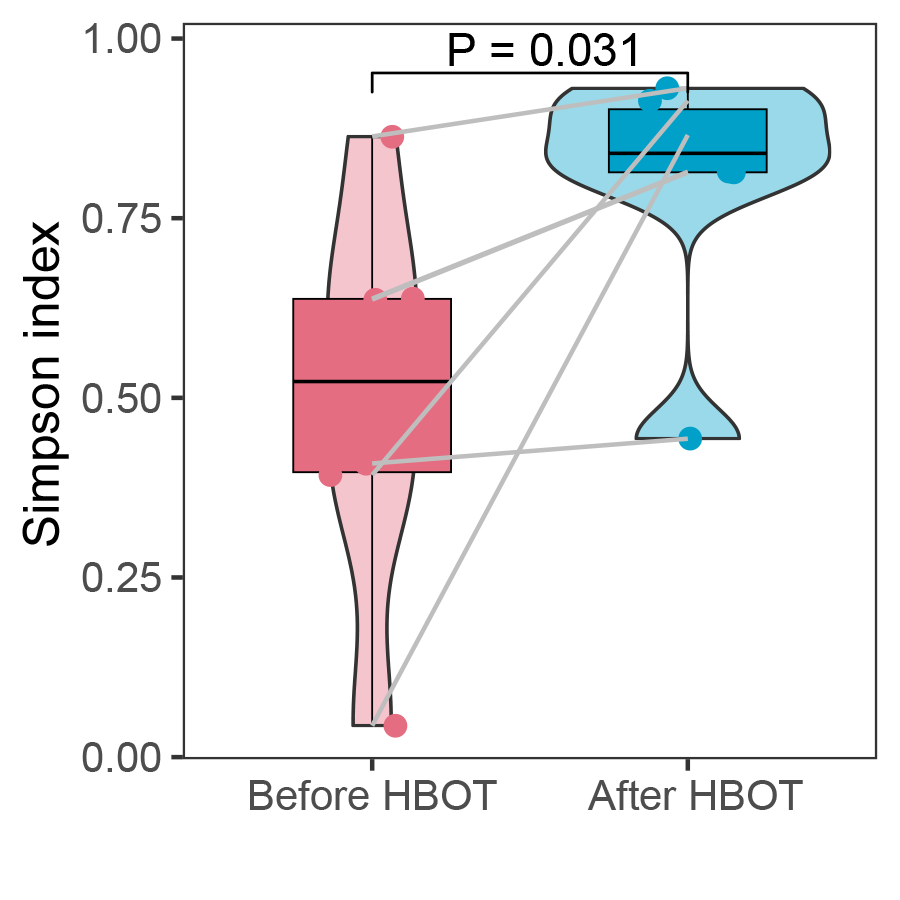
**

**Figure S1.** The boxplot showed the corresponding change of Simpson index in patients with CD before and after HBOT. HBOT, hyperbaric oxygen therapy.


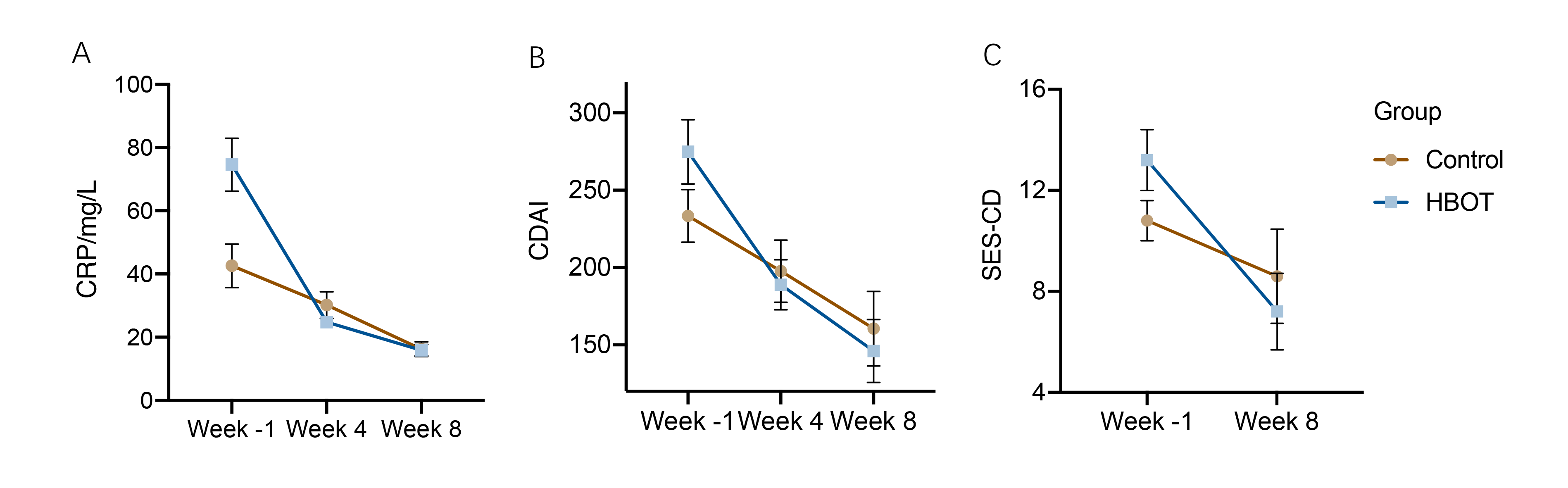


**Figure S2.** Graphs show the changes of CRP level (A), CDAI (B) and SES-CD (C) at different time points (n=10 per group). CRP, C-reactive protein; CDAI, Crohn's disease activity index; SES-CD, simple endoscopic score for Crohn's disease; HBOT, hyperbaric oxygen therapy.


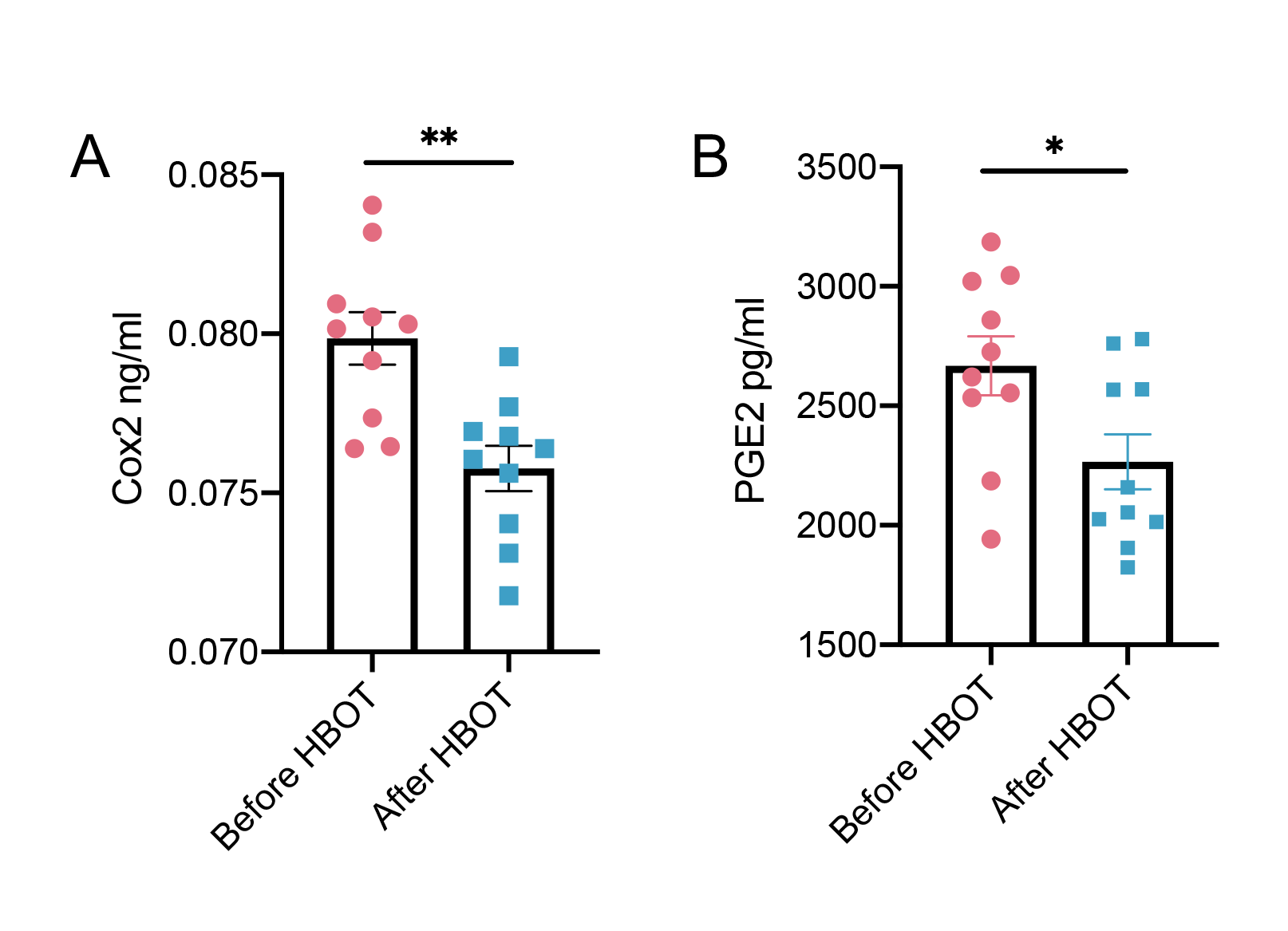


**Figure S3. Comparison of the expression levels of inflammation related proteins in patients before and after hyperbaric oxygen.** ELISA analyses showed the serum levels of COX2, PGE2, before and after 10 sessions of HBOT (n=10 per group). HBOT, hyperbaric oxygen therapy, ** P < 0.01, * P < 0.05

**
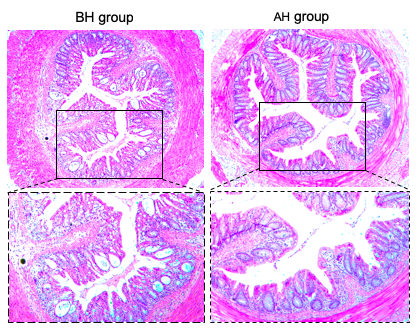
**

**Figure S4. Comparison of DSS colitis mice after 2 weeks of fecal microbiota transplantation.** Representative images of distal colon stained with H&E. Data points represent individual mice.

**
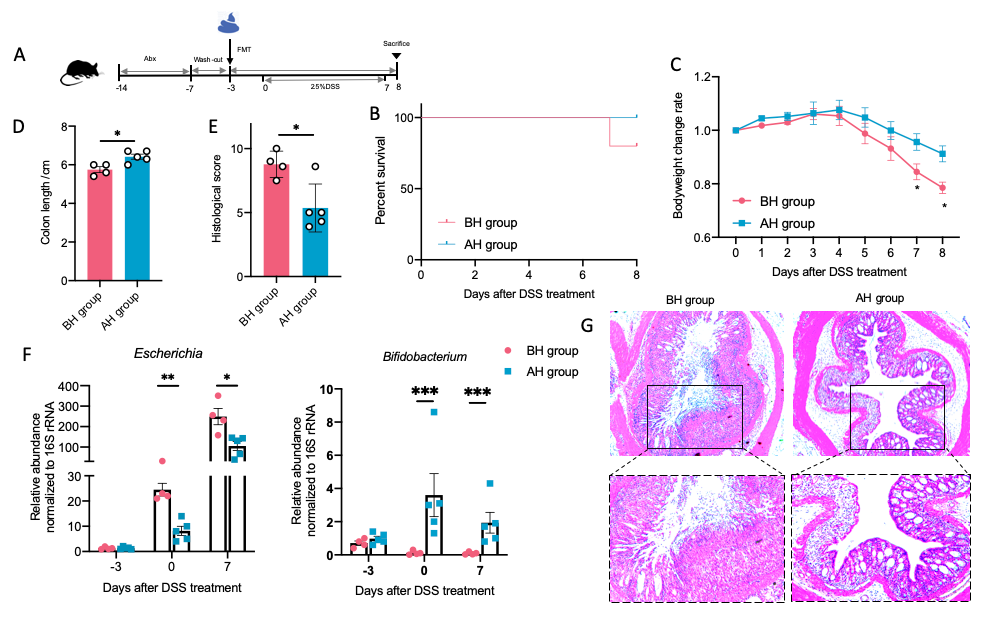
**

**Figure S5. Fecal microbiota transplantation from gut microbiota after HBOT alleviates colitis in female mice.** (A) The experimental design showing an FMT from human donors to mice. (B) Survival curves of two groups of mice (C)Body weights shown as percentage of starting weight and colon length. (D) Colon length. (E) Histological score. (F) Time-course changes in the relative abundance of *Escherichia, Bifidobacterium*. (G) Representative images of distal colon stained with H&E. Data points represent individual mice. All data are represented as means ± SEM. p values were calculated by Student’s t test. For (B), (F) Student’s t test was performed independently at each time point; ∗p < 0.05, ∗∗p < 0.01, and∗∗∗p < 0.001. FMT, Fecal microbiota transplantation; H&E, Hematoxylin and eosin staining; CRP, C-reactive protein.
