# Supplementary figures and images for: Hyperbaric oxygen therapy ameliorates intestinal and systematic inflammation by modulating dysbiosis of the gut microbiota in Crohn’s disease
Source: J Transl Med. 2024 May 30;22:518. doi: 10.1186/s12967-024-05317-1 (PMC11137967; doi:10.1186/s12967-024-05317-1)

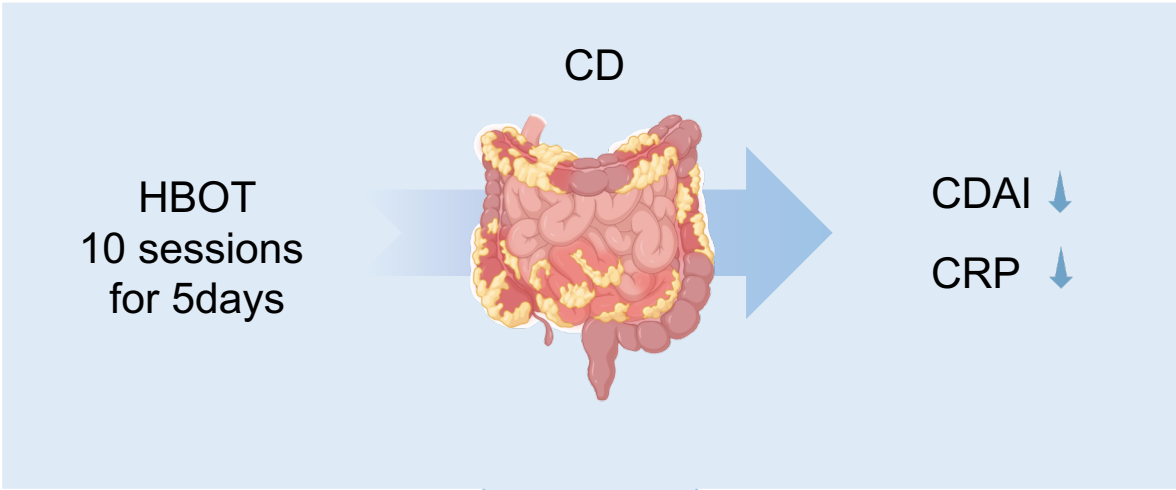

Microbiota alteration

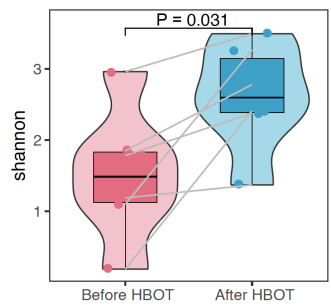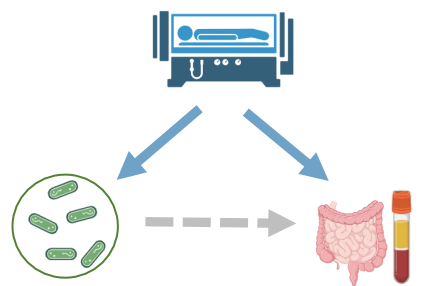

Omics analysis

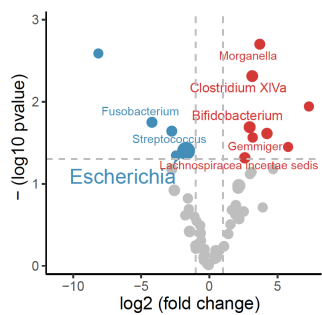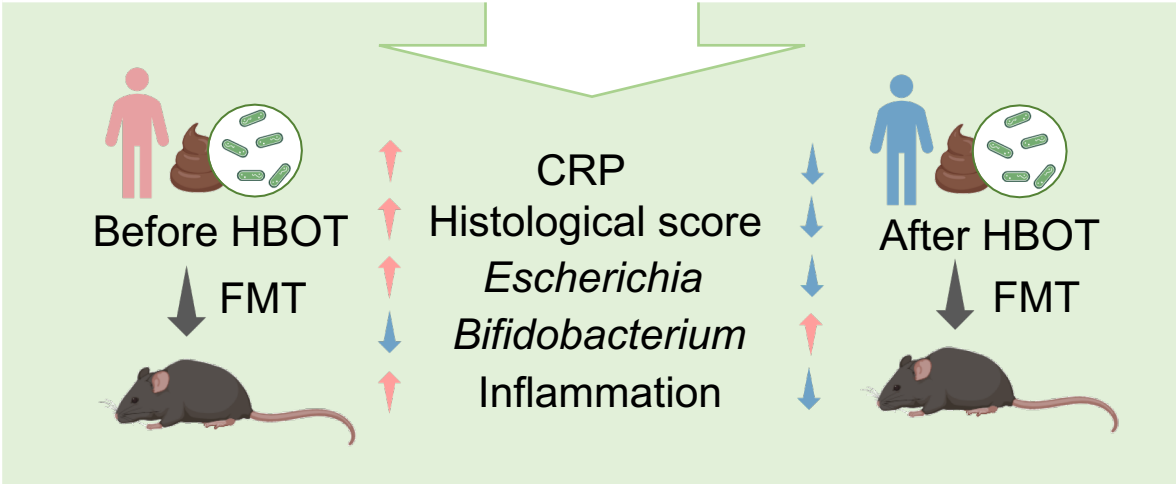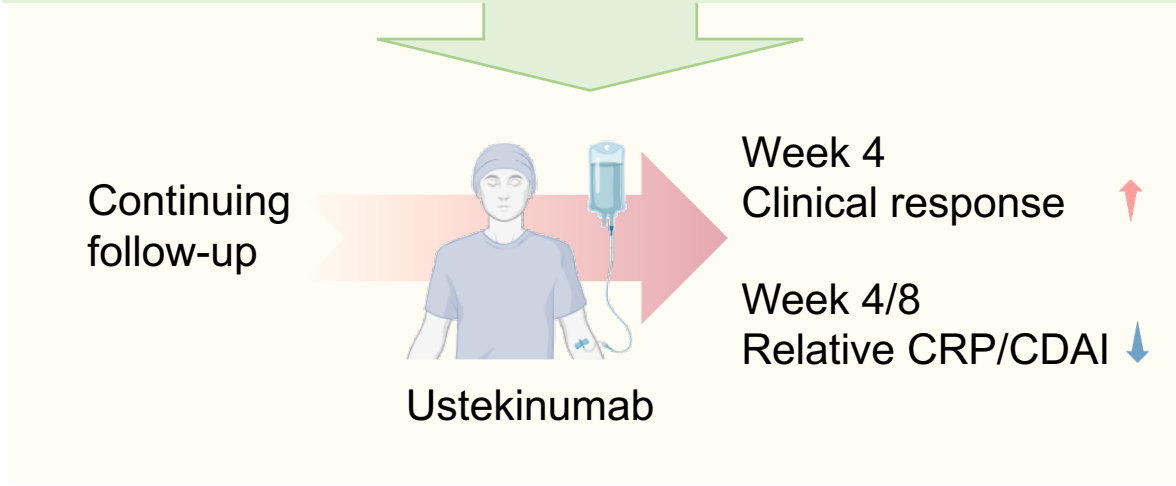

Supplement: Supplementary file 2 — Supplementary Material 2 [file 12967_2024_5317_MOESM2_ESM.pdf]
